# The anti-erbB3 antibody MM-121/SAR256212 in combination with trastuzumab exerts potent antitumor activity against trastuzumab-resistant breast cancer cells

**DOI:** 10.1186/1476-4598-12-134

**Published:** 2013-11-11

**Authors:** Jingcao Huang, Shuiliang Wang, Hui Lyu, Bo Cai, XiaoHe Yang, Jianxiang Wang, Bolin Liu

**Affiliations:** 1 Department of Pathology, School of Medicine, University of Colorado Anschutz Medical Campus, MS-8104, 12801 E. 17th Ave., Aurora, CO 80045, USA; 2 State Key Laboratory of Experimental Hematology, Institute of Hematology and Blood Disease Hospital, 288 Nanjing Road, Tianjin 300020, China; 3 Julius L. Chambers Biomedical/Biotechnology Research Institute, North Carolina Central University, Kannapolis, NC, USA; 4 Fujian Key Laboratory of Transplant Biology, Fuzhou General Hospital, Xiamen University, Fuzhou, Fujian, China

**Keywords:** MM-121, SAR256212, erbB3, erbB2, Trastuzumab resistance, Breast cancer

## Abstract

**Background:**

Elevated expression of erbB3 receptor has been reported to induce resistance to therapeutic agents, including trastuzumab in erbB2-overexpressing breast cancer. Our recent studies indicate that erbB3 interacts with both erbB2 and IGF-1 receptor to form a heterotrimeric complex in trastuzumab-resistant breast cancer cells. Herein, we investigate the antitumor activity of MM-121/SAR256212, a fully human anti-erbB3 antibody (Ab), against two erbB2-overexpressing breast cancer cell lines resistant to trastuzumab.

**Methods:**

MTS-based proliferation assays were used to determine cell viability upon treatment of trastuzumab and/or MM-121/SAR256212. Cell cycle progression was examined by flow cytometric analysis. Western blot analyses were performed to determine the expression and activation of proteins. Tumor xenografts were established by inoculation of the trastuzumab-resistant BT474-HR20 cells into nude mice. The tumor-bearing mice were treated with trastuzumab and/or MM-121/SAR256212 via i.p injection to determine the Abs’ antitumor activity. Immunohistochemical analyses were carried out to study the Abs’ inhibitory effects on tumor cell proliferation and induction of apoptosis *in vivo*.

**Results:**

MM-121 significantly enhanced trastuzumab-induced growth inhibition in two sensitive and two resistant breast cancer cell lines. MM-121 in combination with trastuzumab resulted in a dramatic reduction of phosphorylated erbB3 (P-erbB3) and Akt (P-Akt) in the *in vitro* studies. MM-121 combined with trastuzumab did not induce apoptosis in the trastuzumab-resistant cell lines under our cell culture condition, rather induced cell cycle G1 arrest mainly associated with the upregulation of p27^kip1^. Interestingly, in the tumor xenograft model established from the trastuzumab-resistant cells, MM-121 in combination with trastuzumab as compared to either agent alone dramatically inhibited tumor growth correlated with a significant reduction of Ki67 staining and increase of cleaved caspase-3 in the tumor tissues.

**Conclusions:**

The combination of MM-121 and trastuzumab not only inhibits erbB2-overexpressing breast cancer cell proliferation, but also promotes the otherwise trastuzumab-resistant cells undergoing apoptosis in an *in vivo* xenografts model. Thus, MM-121 exhibits potent antitumor activity when combined with trastuzumab under the studied conditions. Our data suggest that further studies regarding the suitability of MM-121 for treatment of breast cancer patients whose tumors overexpress erbB2 and become resistant to trastuzumab may be warranted.

## Background

Amplification and/or overexpression of e*rbB2* (or *HER2/neu*) occur in approximately 25% of invasive breast cancer and are significantly associated with a worse prognosis for breast cancer patients [1-3]. As an erbB2-targeted therapy, trastuzumab (also known as Herceptin, a humanized monoclonal antibody (Ab) against erbB2) has been approved by FDA and demonstrated significant activity in the treatment of breast cancer patients with erbB2-overexpressing (erbB2+) tumors [4-6]; however, both primary (*de novo*) and acquired resistances to trastuzumab are common and currently represent a significant clinical problem [7-9]. Thus, identification of novel therapeutic strategies/agents to overcome trastuzumab resistance is vital to improve the survival of breast cancer patients whose tumors overexpress erbB2.

Studies on the underlying mechanisms suggest that increased resistance to therapeutic agents is one of the major mechanisms by which erbB2 contributes to breast tumorigenesis [10]. Nonetheless, erbB2 does not act in isolation. It often interacts with other receptor tyrosine kinases (RTKs), such as erbB3, to activate the oncogenic signaling, like PI-3K/Akt pathway, in breast cancers [11]. Co-expression of erbB3 and erbB2 is frequently observed in breast cancers [12] and breast cancer cell lines [13], and erbB3 plays an important role in breast cancer development driven by *erbB2* amplification/overexpression [14]. It has been shown that erbB3 serves as a critical co-receptor of erbB2, and its expression is a rate-limiting factor for erbB2-induced breast cancer cell survival and proliferation [14,15]. Unlike the widely studied erbB2 and EGFR in human cancers, there has been relatively less emphasis on erbB3 as a molecular target for cancer treatment. Currently used erbB2-targeted therapies in clinic can be divided into two strategies: blocking Ab, such as trastuzumab targeting erbB2; and tyrosine kinase inhibitor, such as lapatinib against both EGFR and erbB2. For the erbB3 receptor, because of its lack of or low kinase activity [16,17], targeting of erbB3 with a monoclonal Ab is the only strategy currently under preclinical investigation [18,19] and clinical studies in patients with advanced solid tumors (http://www.clinicaltrials.gov). Recent studies have also identified bispecific Abs dual-targeting of EGFR/erbB3 [20] or erbB2/erbB3 [21], that exhibit potent antitumor activities in laboratory studies. In addition, the erbB3 inhibitors based on a novel biologic scaffold termed a surrobody have been developed and show inhibitory effects on tumor cell proliferation *in vitro* and *in vivo*[22]. MM-121/SAR256212 is a fully human anti-erbB3 monoclonal IgG2 Ab being co-developed by Merrimack Pharmaceuticals and Sanofi. It inhibits ligand-induced dimerization of erbB3 and erbB2 and subsequently inactivates the downstream signaling. MM-121 has been demonstrated to exert antitumor activity in preclinical models of human cancers, including erbB2+ breast cancer [18,19]. However, whether MM-121 holds potential to overcome trastuzumab resistance and enhance trastuzumab-mediated growth inhibition in erbB2+ breast cancer cells remains unclear.

Mechanistic studies implicate the function of erbB3 as a major cause of treatment failure in human cancers [23]. In the last several years, our laboratory has focused on studying the biologic features of erbB3 receptor in erbB2+ breast cancer, and published a serious of articles indicating that activation of erbB3 signaling, mainly through PI-3K/Akt pathway, is essential for erbB2-induced therapeutic resistance to tamoxifen [24], paclitaxel [25], and trastuzumab [26]. Interestingly, activation of the PI-3K/Akt signaling has been identified as the major determinant of trastuzumab resistance [27]. Indeed, our recent studies with the unique trastuzumab-resistant breast cancer model demonstrate that the erbB3 receptor interacts with both erbB2 and the insulin-like growth factor-1 receptor (IGF-1R) to form a heterotrimeric complex, which mainly activates the PI-3K/Akt signaling and Src kinase and subsequently leads to trastuzumab resistance [26]. We hypothesized that the anti-erbB3 Ab MM-121 can overcome trastuzumab resistance and enhance the efficacy of trastuzumab against erbB2+ breast cancer. In the current study, we investigated the potential of MM-121 in combination with trastuzumab on inducing growth inhibition and/or apoptosis in two trastuzumab-sensitive and two trastuzumab-resistant breast cancer cell lines *in vitro*, and explored their inhibitory effects on the growth of tumor xenografts-derived from a trastuzumab-resistant breast cancer cell line *in vivo*.

## Results

### MM-121 significantly enhances the inhibitory effects of trastuzumab on erbB2+ breast cancer cell lines associated with the inactivation of erbB3/PI-3K/Akt signaling

**Figure 1:**
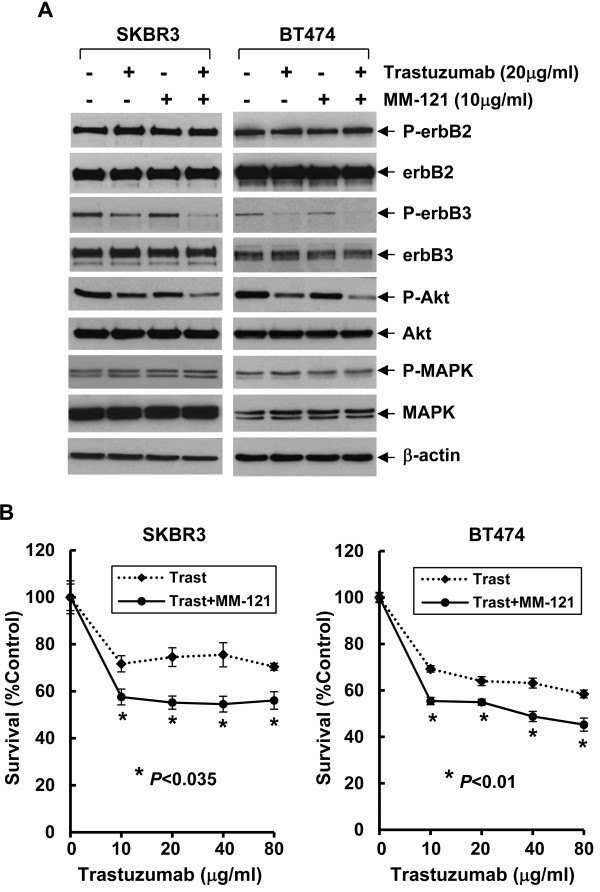
**MM-121 enhances trastuzumab-mediated inactivation of Akt and growth inhibition in two erbB2+ breast cancer cell lines. A**, SKBR3 and BT474 breast cancer cells were untreated or treated with either trastuzumab or MM-121 alone, or their combinations for 24 hrs. Cells were collected and subjected to western blot analyses of P-erbB2, erbB2, P-erbB3, erbB3, P-Akt, Akt, P-MAPK, MAPK, or β-actin. **B**, SKBR3 and BT474 cells were plated onto 96-well plates and incubated at 37°C with 5% CO2. After 24 hrs, the culture medium was replaced with 0.1 ml fresh medium containing 0.5% FBS or the same medium containing the indicated concentrations of trastuzumab in the absence (Trast) or presence (Trast + MM-121) of MM-121 (10 μg/ml) for another 72 hrs. The percentages of surviving cells from each cell line relative to controls, defined as 100% survival, were determined by reduction of MTS. *Bars*, SD. Data show a representative of three independent experiments.

**Figure 2:**
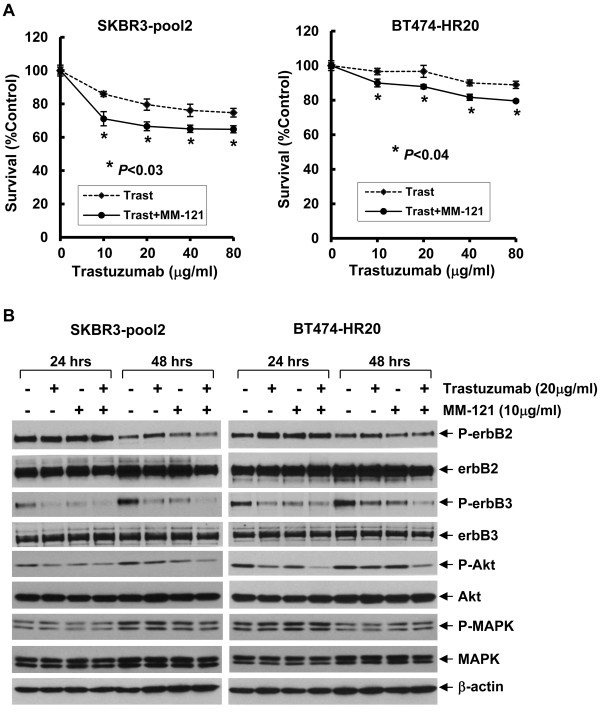
**MM-121 significantly enhances trastuzumab-induced growth inhibition in two otherwise resistant cell lines correlated with inactivation of the erbB3/PI-3K/Akt signaling. A**, SKBR3-pool2 and BT474-HR20 cells were plated onto 96-well plates and incubated at 37°C with 5% CO2. After 24 hrs, the culture medium was replaced with 0.1 ml fresh medium containing 0.5% FBS or the same medium containing the indicated concentrations of trastuzumab in the absence (Trast) or presence (Trast + MM-121) of MM-121 (10 μg/ml) for another 72 hrs. The percentages of surviving cells from each cell line relative to controls, defined as 100% survival, were determined by reduction of MTS. *Bars*, SD. Data show a representative of three independent experiments. **B**, SKBR3-pool2 and BT474-HR20 cells were untreated or treated with either trastuzumab or MM-121 alone, or their combinations for 24 or 48 hrs. Cells were collected and subjected to western blot analyses with the indicated antibodies.

To explore whether the anti-erbB3 Ab MM-121 may enhance the activity of trastuzumab against erbB2+ breast cancers, we investigated the combinatorial effects of MM-121 and trastuzumab on erbB3 signaling and cell proliferation in two erbB2+ breast cancer cell lines (SKBR3 and BT474). The cells were treated with either MM-121 or trastuzumab alone, or their combinations for 24 hrs, and then subjected to western blot analysis. We found that treatment with trastuzumab mainly reduced the levels of phosphorylated erbB3 (P-erbB3) and phosphorylated Akt (P-Akt) in both SKBR3 and BT474 cell lines, whereas MM-121 had no obvious effects on P-erbB3 and P-Akt. However, the combinations of MM-121 and trastuzumab more potently decreased P-erbB3 and P-Akt as compared to trastuzumab alone in SKBR3 cells and to a less extend in BT474 cells (Figure 1A). Neither MM-121 or trastuzumab alone, nor their combinations had significant effects on erbB2 kinase activity, MAPK signaling, and the expression of erbB2/erbB3 receptors (Figure 1A). Cell growth assays revealed that trastuzumab inhibited proliferation of SKBR3 and BT474 cell lines in a dose-dependent manner, consistent with our previous findings [26]. The addition of MM-121 significantly enhanced trastuzumab-mediated growth inhibition in both SKBR3 and BT474 cell lines (Figure 1B). Since activation of the erbB3 signaling plays an important role in the development of trastuzumab resistance [26], we next studied whether MM-121 might overcome the resistance and enhance trastuzumab-mediated growth inhibition in two otherwise resistant breast cancer cell lines. SKBR3-pool2 and BT474-HR20 are trastuzumab-resistant sublines-derived from SKBR3 and BT474 cell lines, respectively [26,28]. While SKBR3-pool2 cells were kindly provided by Dr. Francisco Esteva at MD Anderson Cancer Center [28,29], the BT474-HR20 subline was developed by our laboratory through continuously exposing BT474 cells to trastuzumab in culture for 4 months [26,30]. Indeed, both SKBR3-pool2 and BT474-HR20 cells maintained their resistant phenotype to trastuzumab treatment as compared to their sensitive counterparts (Figure 2A vs Figure 1B). However, the presence of MM-121 significantly enhanced trastuzumab-mediated growth inhibition in both SKBR3-pool2 and BT474-HR20 cell lines (Figure 2A). Further studies on erbB3 activation and the downstream signaling showed that while either MM-121 or trastuzumab alone induced a clear reduction of P-erbB3 and P-Akt and had no significant effects on P-erbB2 and P-MAPK, the combinations of MM-121 and trastuzumab dramatically reduced P-erbB3 and P-Akt in both SKBR3-pool2 and BT474-HR20 cell lines (Figure 2B). Taken together, our data indicate that the erbB3 blocking Ab MM-121 significantly enhances trastuzumab-induced growth inhibition in two erbB2+ breast cancer cell lines and exhibits potential to overcome trastuzumab resistance mainly through inactivation of the erbB3/PI-3K/Akt signaling.

### MM-121 in combination with trastuzumab induces cell cycle G1 arrest in both trastuzumab-sensitive and -resistant breast cancer cell lines

**Figure 3:**
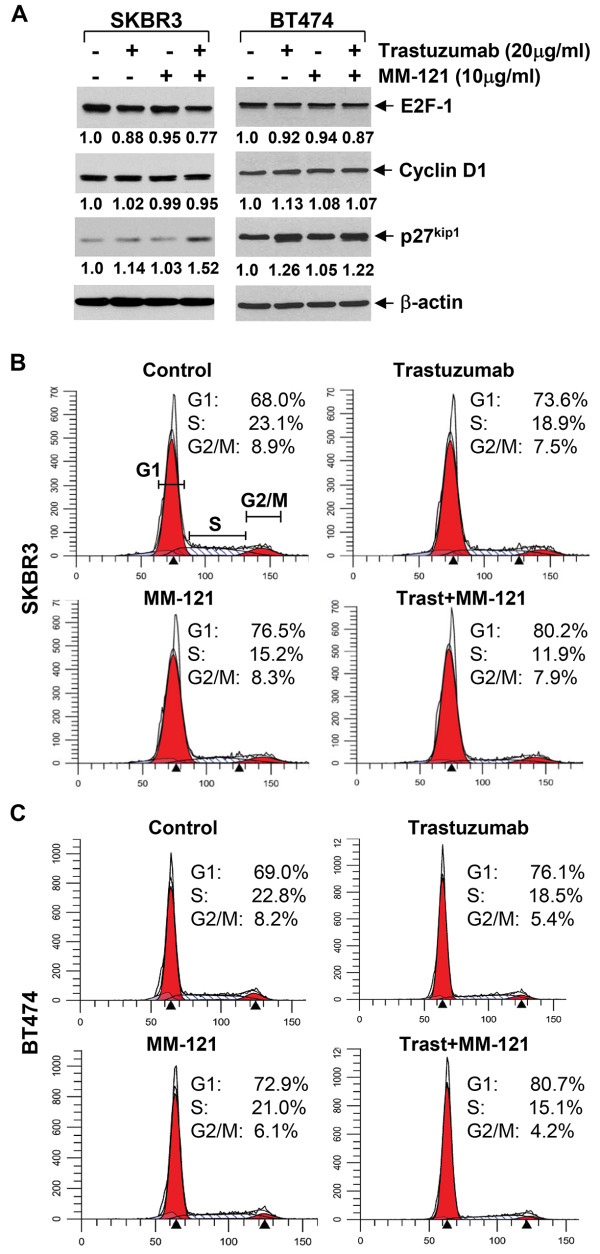
**The addition of MM-121 enhances trastuzumab-induced cell cycle G1 arrest in erbB2+ breast cancer cell lines.** SKBR3 and BT474 cells were untreated or treated with either trastuzumab or MM-121 alone, or their combinations for 24 hrs. **A**, Half of the cells were collected and subjected to western blot analyses with specific antibodies directed against E2F-1, Cyclin D1, p27^kip1^, or β-actin. The densitometry analyses of E2F-1, Cyclin D1, and p27^kip1^ signals were shown underneath, and the arbitrary numbers indicate the intensities of each sample relative to controls, defined as 1.0. **B** &**C**, The other half of the cells were collected for analysis of cell cycle distributions by flow cytometry as described in the Materials and Methods. Data show a representative of three independent experiments.

**Figure 4:**
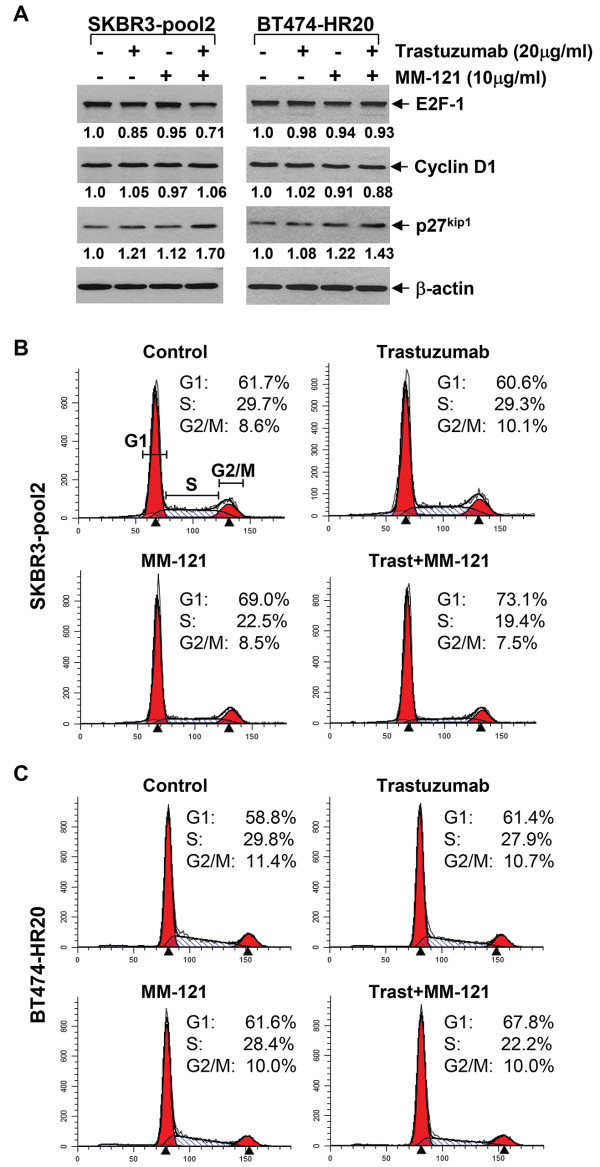
**The combination of MM-121 and trastuzumab induces cell cycle G1 arrest mainly associated with induction of p27**^**kip1 **^**in two trastuzumab-resistant cell lines.** SKBR3-pool2 and BT474-HR20 cells were untreated or treated with either trastuzumab or MM-121 alone, or their combinations for 24 hrs. **A**, Half of the cells were collected and subjected to western blot analyses with specific antibodies directed against E2F-1, Cyclin D1, p27^kip1^, or β-actin. The densitometry analyses of E2F-1, Cyclin D1, and p27^kip1^ signals were shown underneath, and the arbitrary numbers indicate the intensities of each sample relative to controls, defined as 1.0. **B** &**C**, The other half of the cells were collected for cell cycle analysis. Data show a representative of three independent experiments.

To study the molecular mechanism by which MM-121 overcomes trastuzumab resistance and enhances trastuzumab’s efficacy on inhibition of cell proliferation and/or survival in the studied cell lines, we considered the mechanism of action of trastuzumab inducing cell cycle G1 arrest [4,31,32] and thus investigated the combinatorial effects of MM-121 and trastuzumab on the expression levels of several critical molecules participating in G1-S transition and cell cycle progression in erbB2+ breast cancer cell lines. In the trastuzumab-sensitive cells, trastuzumab alone induced a minor reduction of E2F-1 and a slight increase of p27^kip1^ in SKBR3 cells, and it only upregulated p27^kip1^ in BT474 cells (Figure 3A). MM-121 alone did not alter the expression levels of E2F-1 and p27^kip1^ in either cell lines. However, the combinations of trastuzumab and MM-121 clearly increased the levels of p27^kip1^ in both cell lines and led to a minor reduction of E2F-1 in SKBR3 cells (Figure 3A). The expression levels of cyclin D1 were not significantly changed upon treatment with trastuzumab and/or MM-121. Flow cytometry analysis of cell cycle distribution showed that trastuzumab alone increased the cells at G1 phase in both cell lines, whereas MM-121 enhanced G1 population only in SKBR3 cells. Importantly, MM-121 in combination with trastuzumab more dramatically increased the percentage cells at G1 phase and decreased the cells at S phase in both cell lines (Figure 3B), suggesting a further induction of G1 arrest. In the trastuzumab-resistant cells, the expression levels of p27^kip1^ were slightly increased upon treatment with either trastuzumab or MM-121 alone, whereas the combinations of MM-121 and trastuzumab not only upregulated p27^kip1^ in both SKBR3-pool2 and BT474-HR20 cell lines, but also decreased E2F-1 in SKBR3-pool2 cells (Figure 4A). Furthermore, cell cycle analysis confirmed that the combinations of MM-121 and trastuzumab exhibited more potent activity than either agent alone to increase the G1 population and decrease the cells at S phase (Figure 4B). MM-121 and/or trastuzumab had no significant effect on G2-M transition in both trastuzumab-sensitive and -resistant cells (Figures 3B & 4B). While Figures 3B and 4B show the representative data, statistical analyses of G1 population from multiple cell cycle assays were also performed, and we found that the combinations of MM-121 and trastuzumab as compared to trastuzumab alone significantly increased G1 population in SKBR3, SKBR3-pool2, and BT474-HR20 cells (Additional file 1: Figure S1). Additional studies on induction of apoptosis showed that MM-121 and/or trastuzumab did not induce apoptosis in our cell culture condition (data not shown). Collectively, our studies suggest that the combinations of MM-121 and trastuzumab inhibited proliferation of both trastuzumab-sensitive and trastuzumab-resistant breast cancer cells mainly through cell cycle G1 arrest, which was correlated with the upregulation of p27^kip1^ and sometimes a concomitant downregulation of E2F-1.

### The combinations of MM-121 and trastuzumab significantly inhibit growth of tumor xenografts-established from a trastuzumab-resistant breast cancer cell line in nude mice

**Figure 5:**
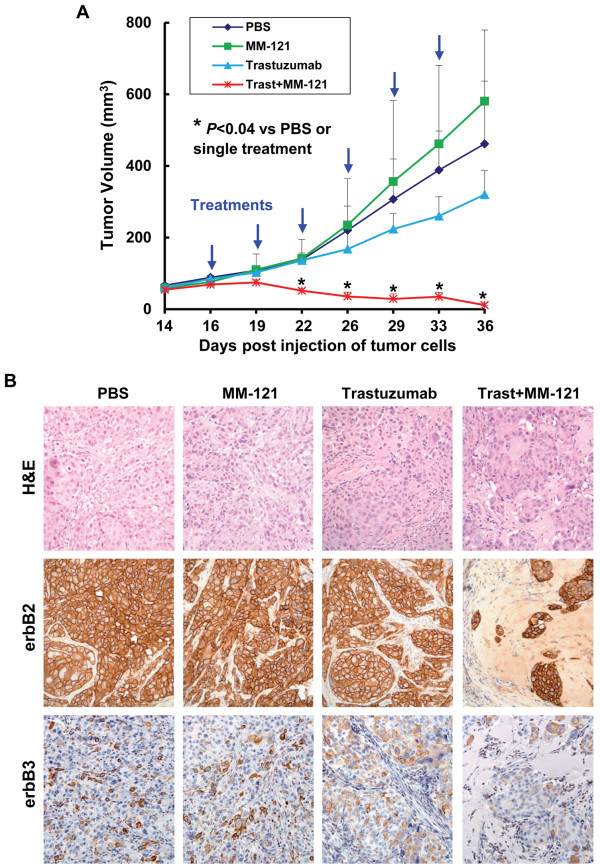
**MM-121 in combination with trastuzumab significantly inhibits *in vivo *growth of tumor xenografts established from BT474-HR20 trastuzumab-resistant breast cancer cells.** BT474-HR20 cells were s.c. injected into nude mice to establish tumor xenografts. The tumor-bearing mice (n = 5) received i.p. injections of PBS, trastuzumab, MM-121, or trastuzumab plus MM-121 as described in the Materials and Methods. After 6 treatments, the mice were euthanized at day 36 post injection of tumor cells, and all tumors were excised for histology and IHC analysis. **A**, The graphs show the tumor growth curves. *Bars*, SD. The combinations of MM-121 and trastuzumab significantly inhibited tumor growth as compared to control or single Ab treatment. **B**, Data show the representative tumors with hematoxylin and eosin (H&E) staining and IHC analysis of erbB2 and erbB3. The residual tumor cells obtained from combinatorial treatments retained similar expression levels of erbB2/erbB3 receptors on the cell membrane.

To further explore whether MM-121 holds potential to overcome trastuzumab resistance in an *in vivo* model for breast cancer treatment, we took advantage of the tumor xenografts model established from the trastuzumab-resistant breast cancer cell line BT474-HR20. There is a general concern that erbB2+ breast cancer cell lines are difficult to form spontaneous xenografts in athymic nu/nu mice [33], and it is not known whether the BT474-HR20 cells would maintain their trastuzumab-resistant phenotype *in vivo*. We first compared the ability of trastuzumab-sensitive and -resistant cells to form tumors in nude mice. We found that the BT474-HR20 cells formed tumors with a shorter latency than BT474 cells, and the tumors established from the resistant cells grew significantly faster than those from the parental cells (Additional file 2: Figure S2A), suggesting the aggressive phenotypes of BT474-HR20 cells. Importantly, the tumors-derived from BT474-HR20 cells were still growing under the treatment of trastuzumab (Additional file 2: Figure S2B), whereas the tumors-derived from BT474 cells were eliminated after three doses of trastuzumab (Additional file 2: Figure S2C). These data suggest that although BT474-HR20 cells were obtained in *in vitro* cell culture condition, they still maintained the trastuzumab-resistant phenotype *in vivo*. We next performed the following *in vivo* experiments with Ab treatment. When BT474-HR20 tumor volumes reached ~65 mm^3^, the nude mice were treated with either PBS (control), or MM-121 or trastuzumab alone, or the combinations of MM-121 and trastuzumab. Treatment with trastuzumab alone resulted in a minor and statistically insignificant inhibition (Figure 5A). It appeared that MM-121 alone had a stimulatory effect on the growth of BT474-HR20 tumor xenograft, although the differences were statistically insignificant. However, this phenomenon was not observed consistently. In our recent publication, MM-121 alone had neither positive nor negative effect on *in vivo* tumor growth of BT474-HR20 cells . More importantly, the combinations of MM-121 and trastuzumab significantly inhibited tumor growth of BT474-HR20 cells (Figure 5A). After 6-time treatments, the remaining tumors from the combinatorial treatment were very small. We did observe tumor regression in the time frame of our experiments. Histology and immunohistochemistry (IHC) assays revealed that treatment with MM-121 or trastuzumab alone did not alter tumor cell morphology and the expression of erbB2/erbB3 receptors (Figure 5B). In contrast, the combinatorial treatment resulted in much less tumor cells remaining, lost tumor architecture, and increased fibroblast cells in the tissues. Nonetheless, the remaining tumor cells maintained a similar expression levels of both erbB2 and erbB3 receptors (Figure 5B), which was consistent with the results of our cell culture studies (Figure 2B).

### MM-121 enhances trastuzumab’s antitumor activity against the otherwise resistant breast cancer cells via induction of both cell growth inhibition and apoptosis *in vivo*

**Figure 6:**
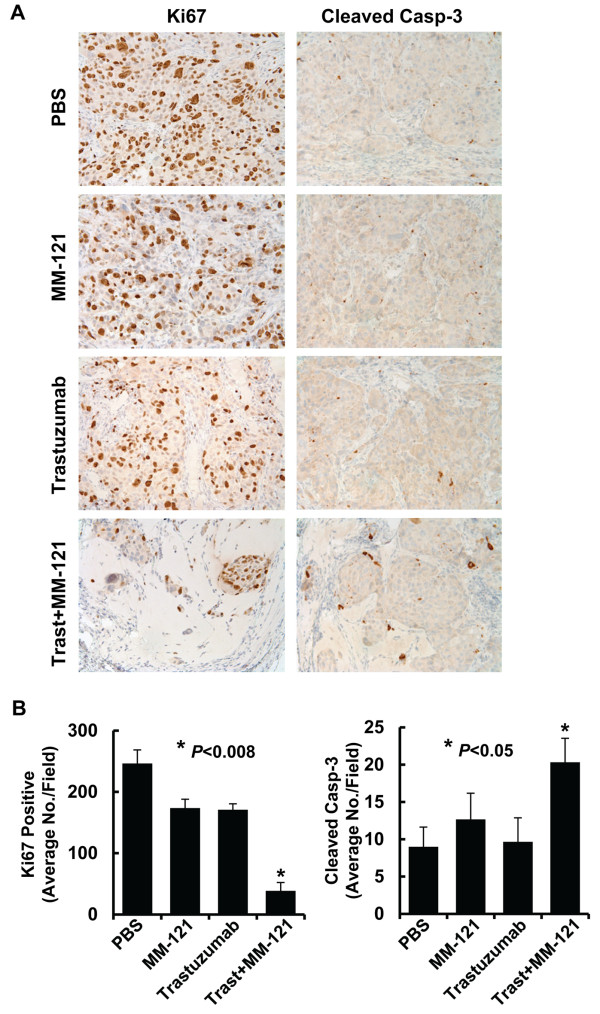
**The combination of MM-121 and trastuzumab significantly inhibits proliferation and induces apoptosis of trastuzumab-resistant BT474-HR20 breast cancer cells *in vivo*.** The tumors obtained from the animal studies described above were evaluated by IHC analysis of Ki67 and cleaved caspase-3. **A**, Data show the representative images of the immunostaining of Ki67 and cleaved caspase-3 (Cleaved Casp-3). **B**, The IHC slides were observed by two independent personnel. The tumor cells with positive staining of Ki67 or cleaved caspase-3 were counted from three randomly selected areas in each slide. The three areas were first identified by scanning the entire slide at ×10 magnification, and then the positive staining cells were counted at ×20 magnification using an Olympus BX40 Microscope. The bar graphs show the average of positive staining cells in each field. *Bars*, SD. The combinatorial treated mice had significantly fewer cells stained positive for Ki67 and more cells stained positive for cleaved caspase-3 than control mice or single Ab treated mice, *P* < 0.008 and *P* < 0.05, respectively.

While our cell culture studies discovered that MM-121 in combination with trastuzumab inhibited proliferation of SKBR3-pool2 and BT474-HR20 cell lines (Figure 2A) without induction of apoptosis (data not shown), we wondered whether the combinations of MM-121 and trastuzumab would have similar effects on the trastuzumab-resistant cells *in vivo*. By utilizing the tumor tissues obtained from the BT474-HR20 xenograft animal studies described above, we then performed IHC studies on the classic cell proliferative marker Ki67 and cleaved caspase-3, an indicative of cells undergoing apoptosis. The mice treated with MM-121 or trastuzumab exhibited a minor reduction in the number of tumor cells with positive staining of Ki67 as compared to the control mice (Figure 6A). Neither MM-121 nor trastuzumab induced caspase-3 cleavage in the tumor tissues. However, the mice treated with both MM-121 and trastuzumab showed a dramatic reduction in the number of tumor cells with positive staining of Ki67 and a significant increase of the tumor cells with cleaved caspase-3 (Figure 6 and Additional file 3: Figure S3). These data indicate that MM-121 enhances trastuzumab’s antitumor activity against the otherwise resistant breast cancer cells via induction of both cell growth inhibition and apoptosis in this *in vivo* model.

## Discussion

As a unique member of the erbB receptor family, erbB3 has long been considered an inactive “pseudokinase” [16,34]. A recent study suggests that erbB3 has weak kinase activity that can trans-autophosphorylate its intracellular region [17]. In order to fully transduce cell signaling, however, erbB3 has to be phosphorylated by its interactive partners, of these, erbB2 is the most important one [35]. It has been well-documented that activation of the erbB3 signaling plays a pivotal role in the development of erbB2+ breast cancer [14,15], castration-resistant prostate cancer [36], platinum resistant/refractory ovarian cancer [37,38], and etc. Nonetheless, no erbB3-targeted therapy has been approved for cancer treatment. MM-121 is an erbB3 blocking Ab that is being actively investigated, mainly in combination with chemotherapy, in clinical trials of cancer patients with solid tumors, such as advanced non-small cell lung cancer, colorectal cancer, squamous cell head & neck cancer, platinum resistant/refractory ovarian cancer (http://www.clinicaltrials.gov/ct2/results?term=mm-121). In breast cancer, MM-121’s therapeutic potential is being tested in patients with ER and/or PR positive and erbB2 negative breast cancers in combination with the aromatase inhibitor exemestane, and in patients with triple negative or erbB2 negative breast cancers in combination with paclitaxel. To date, no clinical study has been initiated to test MM-121’s activity in breast cancer patients with erbB2+ tumors, particularly those become resistant to trastuzumab. Here, we demonstrated that MM-121 significantly enhanced trastuzumab-mediated growth inhibition in two sensitive and two resistant breast cancer cell lines. More importantly, the studies using a specific tumor xenograft model further proved that MM-121 exerted potent activity to overcome trastuzumab resistance in that *in vivo* model. Thus, our data provide a strong basis to explore the therapeutic potential of MM-121 in combination with trastuzumab in erbB2+ breast cancer patients resistant to trastuzumab.

Our previous studies showed that the mechanism of trastuzumab resistance in SKBR3-pool2 and BT474-HR20 cells was due to the formation of a heterotrimeric complex consisting of erbB2, erbB3, and IGF-1R [26]. We discovered that the expression of both erbB3 and IGF-1R was critical for maintaining trastuzumab-resistant phenotype, since specific knockdown of either erbB3 or IGF-1R significantly abrogated the resistance in SKBR3-pool2 and BT474-HR20 cells [26]. The data presented here indicated that inhibiting erbB3, but retaining its expression, also re-sensitized the resistant cells to the treatment of trastuzumab in our *in vitro* (Figure 2) and *in vivo* (Figure 5) models. It is not clear, however, whether inactivation of erbB3 by MM-121 overcomes trastuzumab resistance via disrupting the heterotrimerization of erbB2/erbB3/IGF-1R. At this moment, the molecular basis of this heterotrimerization remains unknown. We speculate that long-term exposure of SKBR3 or BT474 cells to trastuzumab may induce expression of the ligands for erbB3 (heregulin, HRG) and IGF-1R (IGF-I and/or IGF-II), which could subsequently recruit all three RTKs together to form the unique heterotrimeric complex. Since MM-121 inhibits ligand-induced dimerization between erbB3 and erbB2 [18,19], it may also interfere with the heterotrimeric complex consisting of erbB2, erbB3, and IGF-1R in SKBR3-pool2 and BT474-HR20 cells and thus overcome the resistance. However, detailed studies are warranted to test this hypothesis.

The combinations of MM-121 and trastuzumab inhibited proliferation of two sensitive and two resistant breast cancer cell lines *in vitro*; however, they induced both growth inhibition and apoptosis *in vivo*. This cell killing effects may be attributed to the enhanced antibody-dependent cell-mediated cytotoxicity (ADCC) by natural killer (NK) cells. Abundant evidence demonstrates that one of the major mechanisms of action of trastuzumab is through its IgG1 humanized Fc portion to activate ADCC via host’s innate immune system [32]. In addition, cellular adaptive immune response also plays an important role in the clinical efficacy of trastuzumab [39]. Novel strategies that enhance ADCC effectors, such as NK cells, are sought to improve trastuzumab efficacy. A recent study reported exciting data indicating that stimulation of NK cells with a CD137-specific Ab significantly enhanced trastuzumab-mediated cell killing in both sensitive and resistant cell lines *in vitro* and *in vivo*[40]. In our case, although MM-121 itself cannot trigger ADCC, because of its IgG2 isotype [41], it is possible that inactivation of erbB3 with MM-121 may increase trastuzumab’s binding efficiency to the tumor xenografts-established from BT474-HR20 cells, and subsequently enhance trastuzumab-mediated ADCC.

Activation of erbB3 generally signals through PI-3K/Akt, MEK/MAPK, Jak/Stat pathways, and Src kinase to modulate many downstream regulators that play a pivotal role in maintaining malignant phenotype, including cell survival, resistance, angiogenesis, and invasion [16,42]. Our data showed that treatment of certain erbB2+ breast cancer cell lines with MM-121 resulted in a dramatic inhibition on PI-3K/Akt signaling, the major determinant of trastuzumab resistance in breast cancer [27]. However, it is not known whether MM-121 may potentially abrogate resistance to lapatinib, another erbB2-targeted therapy to treat metastatic breast cancer that has progressed after trastuzumab-based therapy [43]. Lapatinib and trastuzumab may not share common mechanism of resistance, as lapatinib has activity in trastuzumab-resistant breast cancer [44-47]. Some studies show that lapatinib exerts antitumor activity in a PTEN independent manner [48], whereas others report that loss of PTEN and the resulting activation of PI-3K/Akt signaling lead to lapatinib resistance [49]. Thus, it will be very interesting, and may have clinical implications, to study if the combinations of MM-121 and lapatinib may synergistically or additively induce growth inhibition and/or apoptosis in BT474-HR20 and SKBR3-pool2 cells. In addition, activation of the erbB2/erbB3/PI-3K/Akt signaling also results in resistance to hormonal therapy [50] and chemotherapy [51] in breast cancer treatment. We have reported that elevated expression of erbB3 confers paclitaxel resistance in erbB2+ breast cancer cells via a PI-3K/Akt-dependent mechanism [25]. Because MM-121 mainly inhibits activation of erbB3 and Akt (Figures 1 & 2), it is conceivable to hypothesize that MM-121 may abrogate erbB3 signaling-mediated resistance to paclitaxel as well. Indeed, we have discovered that MM-121 is able to overcome paclitaxel resistance and enhance paclitaxel-induced apoptosis in the otherwise resistant breast cancer cell lines. The manuscript containing those data is submitted separately.

## Conclusions

MM-121 significantly enhances trastuzumab-induced growth inhibition in erbB2+ breast cancer cell lines. MM-121 is active to overcome trastuzumab resistance in the studied *in vitro* and *in vivo* models. When combined with trastuzumab, MM-121 mainly inhibits proliferation, without induction of apoptosis, via cell cycle G1 arrest *in vitro.* However, their combinatorial *in vivo* antitumor activity against the trastuzumab-resistant breast cancer cells is attributed to induction of both growth inhibition and apoptosis. Our data support further studies to explore the therapeutic potential of MM-121 in combination with trastuzumab in breast cancer patients whose tumors overexpress erbB2 and become resistant to trastuzumab.

## Methods

### Reagents and antibodies

MM-121 was kindly provided by Merrimack Pharmaceuticals, Inc. (Cambridge, MA). Trastuzumab (Herceptin®, Genentech, South San Francisco, CA) was obtained from University of Colorado Hospital pharmacy. Antibodies used for western blots were as follows: erbB2 (EMD Chemicals, Inc., Gibbstown, NJ); erbB3 and P-erbB2 (Tyr1248) (LabVision Corp., Fremont, CA); P-erbB3 (Tyr1289), P-MAPK (Thr202/Tyr204), MAPK, P-Akt (Ser473), and Akt (Cell Signaling Technology, Inc., Beverly, MA); Cyclin D1 (M-20), E2F1 (KH95), and p27^kip1^ (F-8) (Santa Cruz Biotechnology, Inc., Santa Cruz, CA); and β-actin (Sigma Co., St. Louis, MO). All other reagents were purchased from Sigma unless otherwise specified.

### Cells and cell culture

Human breast cancer cell lines SKBR3 and BT474 were obtained from the American Type Culture Collection (ATCC, Manassas, VA). The trastuzumab-resistant sublines SKBR3-pool2 and BT474-HR20, derived from SKBR3 and BT474, respectively, were described previously [26]. All cell lines were maintained in DMEM/F-12 (1:1) medium (Sigma) containing 10% fetal bovine serum (Sigma), and cultured in a 37°C humidified atmosphere containing 95% air and 5% CO2 and split twice a week.

### Cell proliferation assay

The CellTiter96 AQ nonradioactive cell proliferation kit (Thermo Fisher Scientific Inc., Waltham, MA) was used to determine cell viability as previously described [25,26]. Briefly, cells were plated onto 96-well plates for 24 h, and then grown in either DMEM/F12 medium with 0.5% FBS as control, or the same medium containing different concentrations of trastuzumab in the presence or absence of MM-121, and then incubated for another 72 h. After reading all wells at 490 nm with a microplate reader, the percentages of surviving cells from each group relative to controls, defined as 100% survival, were determined by reduction of MTS.

### Cell cycle analysis

Flow cytometric assays were performed as described previously [52] to define the cell cycle distribution. In brief, cells grown in culture dishes were harvested by trypsinization and fixed with 70% ethanol. Cells were stained for total DNA content with a solution containing 50 μg/ml propidium iodide and 100 μg/ml RNase I in PBS for 30 min at 37°C. Cell cycle distribution was analyzed at the Flow Cytometry Core Facility of University of Colorado Cancer Center with a FACScan flow cytometer (BD Biosciences, San Jose, CA).

### Western blot analysis

Protein expression levels were determined by western blot analysis as described previously [26,30,52]. Equal amounts of total cell lysates were boiled in Laemmli SDS-sample buffer, resolved by SDS-PAGE, transferred to nitrocellulose membrane (Bio-Rad Laboratories, Hercules, CA), and probed with the primary antibodies described in the figure legends. After the blots were incubated with horseradish peroxidase-labeled secondary antibody (Jackson ImmunoResearch Laboratories, West Grove, PA), the signals were detected using the enhanced chemiluminescence reagents (GE Healthcare Bio-Sciences Corp., Piscataway, NJ).

### Immunohistochemistry

Five micron thick paraffin sections were deparaffinized, antigens unmasked and immunohistochemically stained for Ki67 (Thermo Fisher Scientific; rabbit monoclonal SP6; cat# RM-9106-SO; dilution 1:500 in TBST + 1% BSA w/v), cleaved Caspase-3 (Cell Signaling Technology; rabbit polyclonal; cat#: 9661, 1:1000 in TBST + 1% BSA w/v), erbB2 (EMD Chemicals; mouse monoclonal 96G; cat#OP14T; dilution 1:500 in TBST + 1% BSA w/v), and erbB3 (Spring Bioscience, Pleasanton, CA; rabbit monoclonal SP71; cat# M3710; dilution 1:200 in TBST + 1% BSA w/v). The specificity of all antibodies has been confirmed by both positive and negative controls. For erbB2 and erbB3, SKBR3 cells were used as a positive control. For Ki67 and cleaved caspase-3, the human tonsil tissues were used a positive control. All the negative controls were performed with the same cells/tissues without addition of the primary antibodies.

Ki67 and cleaved caspase-3 antigens were revealed in pH 9.5 BORG solution (Biocare Medical, Concord, CA) for 5 min at 125°C (22 psi; Decloaking chamber, Biocare). ErbB2 required modest retrieval in 10 mmol/L sodium citrate for 5 min at 125°C in the Decloaking chamber. ErbB3 required retrieval in Cell Conditioner 1 (standard retrieval time, Ventana). Immunodetection of Ki67, cleaved Caspase-3 and erbB2 was performed on the NexES stainer (Ventana Medical Systems, Tucson, AZ) at an operating temperature of 37°C. Ki67 and cleaved caspase-3 antibodies were incubated for 32 min and detected with a modified I-VIEW DAB (Ventana) detection kit. The I-VIEW secondary antibody and enzyme were replaced with a species specific secondary antibody (biotinylated goat anti-rabbit; 1:75; cat# 111-065-144; Jackson ImmunoResearch; 8 min) and streptavidin-horseradish (SA-HRP; 1:50; cat# SA-5004; DAKO Cytomation, Carpinteria, CA; 8 min). ErbB2 was incubated for 32 min and detected with the standard I-VIEW detection. ErbB3 was incubated for 32 min and detected with a modified I-VIEW DAB kit in which the secondary antibody was replaced with Rabbit ImmPress (Vector Labs; Burlingame, CA; cat# MP-7401; 8 minutes at 37°C) and enzyme was replaced with Rabbit ImmPress (Diluted 1:1 in PBS pH 7.6; 8 minutes at 37°C). Sections were sequentially blocked for 10 min in 3% hydrogen peroxide (v/v) and 30 min in Rodent Block M (Biocare, cat# RBM961), followed by primary antibody incubation for 30 min and 30 min in polymer. Antibody complexes were visualized with IP Flex DAB (Biocare; cat# IPK5010 G80; 4.5%). All sections were counterstained in Mayer’s hematoxylin for 2 min, nuclei blued in 1% ammonium hydroxide (v/v), dehydrated in graded alcohols, cleared in xylene and coverglass mounted using synthetic resin.

### Tumor xenograft model

Athymic nu/nu mice (Harlan Laboratories, Inc., Indianapolis, IN) were maintained in accordance with the Institutional Animal Care and Use Committee (IACUC) procedures and guidelines. Eight ×10^6^ BT474-HR20 cells were suspended in 100 μL of PBS, mixed with 50% Matrigel (BD Biosciences) and injected subcutaneously (S.C.) into the flanks of 5-week-old female mice. Tumor formation was assessed by palpation and measured with fine calipers three times a week. Tumor volume was calculated by the formula: volume = (length × width^2^)/2, where length was the longest axis and width the measurement at a right angle to the length, and followed by statistical analysis as we described previously [52]. When tumors reach ~65 mm^3^, mice were randomly assigned to four groups (*n* = 5): 1) control group-mice received intraperitoneally (i.p) injection of 100 μl PBS only; 2) mice received i.p. injection of trastuzumab (10 mg/kg) in 100 μl PBS twice a week; 3) mice received i.p. injection of MM-121 (10 mg/kg) in 100 μl PBS twice a week; 4) mice received i.p. injection of trastuzumab (10 mg/kg) and MM-121 (10 mg/kg) in 100 μl PBS twice a week. The animals’ health status was monitored daily for weight loss or for signs of altered motor while in their cages. At the end of study, mice were euthanized according to approved IACUC protocol. Tumors from all animals were excised and embedded in paraffin for immunohistochemical analyses.

### Statistical analysis

Statistical analyses of the experimental data were performed using either a two-sided t test or ANOVA for each time point followed by post-hoc testing between groups. Significance was set at a P value of <0.05. All statistical analyses were conducted with the software StatView v5.1 from SAS Institute Inc., Cary, NC.

## Abbreviations

MBC: Metastatic breast cancer; RTK: Receptor tyrosine kinase; ER: Estrogen receptor; PR: Progestrone receptor; EGFR: Epidermal growth factor receptor; HRG: Heregulin; IGF-I: Insulin-like growth factor-I; IGF-1R: IGF-I receptor; PTEN: Phosphatase and tensin homolog; PI-3K: Phosphoinositide 3-kinase; MAPK: Mitogen-activated protein kinase; ADCC: Antibody-dependent cell-mediated cytotoxicity; NK: Natural killer; IHC: Immunohistochemistry; ELISA: Enzyme-linked immunosorbent assay; MTS: 3-(4,5-dimethylthiazol-2-yl)-5-(3-carboxymethoxyphenyl)-2-(4-sulfophenyl)-2H-tetrazolium,inner salt.

## Competing interests

The authors declare that they have no competing interests.

## Authors’ contributions

The authors’ contributions to this research work are reflected in the order shown, with the exception of JW and BL who supervised the research and finalized the report. JH and SW carried out the majority of the *in vitro* studies and all of the *in vivo* experiments. HL generated the data of cell cycle analysis. JH and BC performed IHC studies and quantified the immunostaining. JW and BL drafted the manuscript. XY generated the BT474-HR20 resistant cell line and maintained its resistant phenotype in cell culture. XY, JW, and BL conceived of the study, and participated in its design and coordination. All authors read and approved the final manuscript.

## Supplementary Material

Additional file 1: Figure S1 Combinations of trastuzumab and MM-121 significantly induced cell cycle G1 arrest in both trastuzumab-sensitive and -resistant breast cancer cells. SKBR3, BT474, SKBR3-pool2, or BT474-HR20 cells were untreated or treated with either trastuzumab (20 μg/ml) or MM-121 (10 μg/ml) alone, or their combinations for 24 hrs. All the cells were collected for analysis of cell cycle distributions by flow cytometry as described in the Methods. The bar graphs show the percentages of the cells at G1 phase for each sample relative to controls, defined as 100%. A, The combinations of trastuzumab and MM-121 as compared to trastuzumab significantly induced G1 arrest in SKBR3 cells. There was no significant difference between these two treatments in BT474 cells, which was very sensitive to the treatment of trastuzumab alone. B, The combinations of trastuzumab and MM-121 as compared to trastuzumab significantly induced G1 arrest in both SKBR3-pool2 and BT474-HR20 cells. *Bars*, SD. Statistical analyses were performed using data from three independent experiments. Click here for file

Additional file 2: Figure S2 Trastuzumab-resistant breast cancer cells show significant growth advantage as compared to their sensitive counterpart and retain their resistant phenotype *in vivo*. A, BT474 or BT474-HR20 cells were injected s.c into the flanks of 5-week-old female nude mice. Mice were checked for tumor formation three times per week. Tumor volume was calculated by the formula: *volume = (length × width*^*2*^*)/2*, and expressed as cubic millimeters. B & C, When tumor volumes reached ~65 mm^3^, the animals were treated with either control (PBS) or trastuzumab (20 mg/kg) four times. Tumors-derived from BT474-HR20 cells were still growing even in the presence of trastuzumab (B), whereas the tumors-derived from BT474 cells were no longer detectable after three doses of trastuzumab (C). Tumor volume was expressed as cubic millimeters (mean ± SE; n = 5/group). Click here for file

Additional file 3: Figure S3 Combinations of trastuzumab and MM-121 significantly increase the percentage of positive-staining cells with cleaved caspase-3 *in vivo*. The tumor IHC slides were observed by two independent personnel. The tumor cells with positive staining of cleaved caspase-3 were counted from three randomly selected areas (a total of 360 tumor cells were counted for each area) in each slide. The three areas were first identified by scanning the entire slide at ×10 magnification, and then the positive staining cells were counted at ×20 magnification using an Olympus B×40 Microscope. The bar graphs show the percentage of cells with positive staining for cleaved caspase-3 from each group. The combinatorial treated mice had significantly higher proportion of cells stained positive for cleaved caspase-3 than the control mice or single Ab treated mice, *P* < 0.002. Click here for file
