# Supplementary figures and images for: The anti-erbB3 antibody MM-121/SAR256212 in combination with trastuzumab exerts potent antitumor activity against trastuzumab-resistant breast cancer cells
Source: Mol Cancer. 2013 Nov 11;12:134. doi: 10.1186/1476-4598-12-134 (PMC3829386; doi:10.1186/1476-4598-12-134)

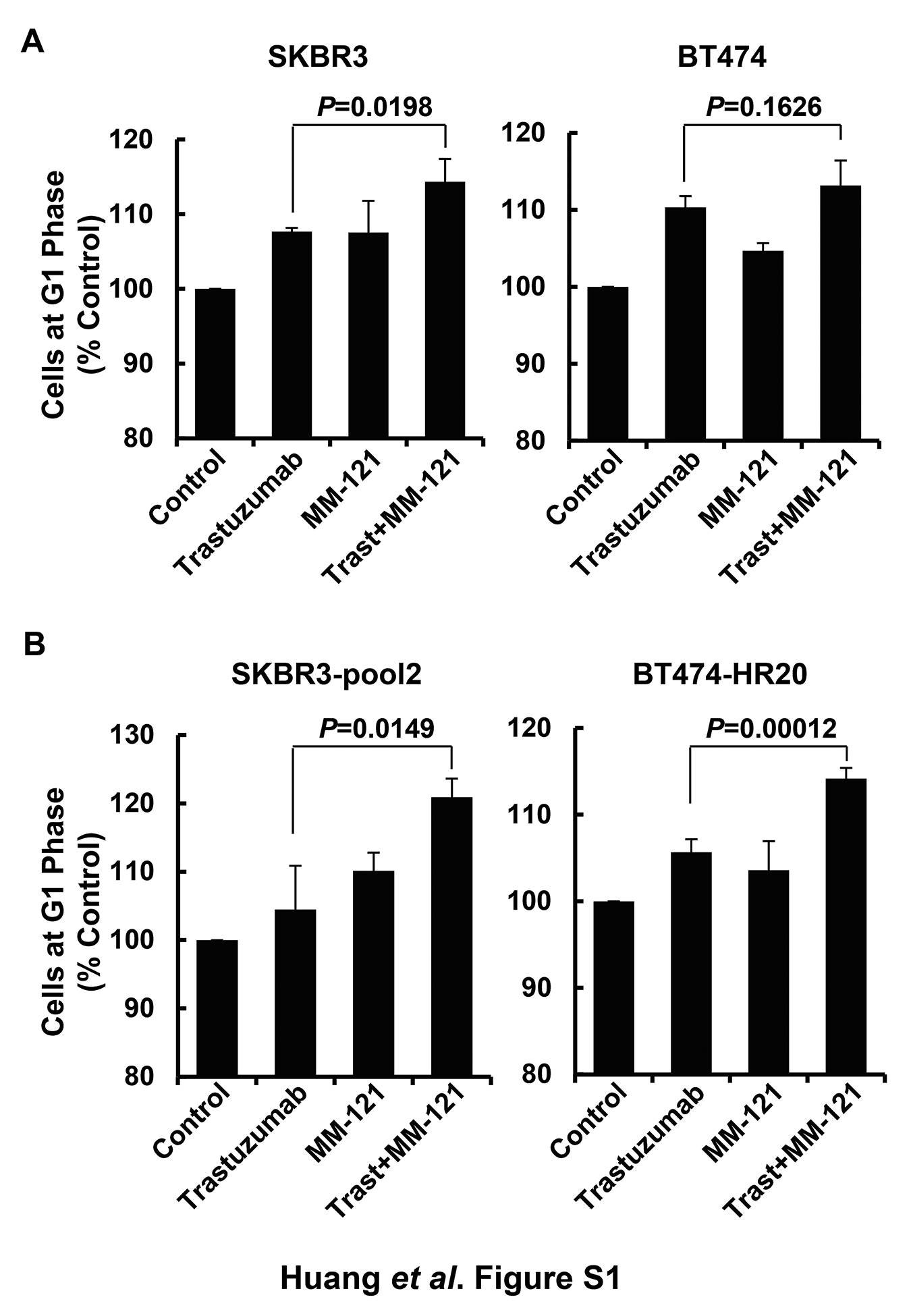

Supplement: Additional file 1: Figure S1 — Combinations of trastuzumab and MM-121 significantly induced cell cycle G1 arrest in both trastuzumab-sensitive and -resistant breast cancer cells. SKBR3, BT474, SKBR3-pool2, or BT474-HR20 cells were untreated or treated with either trastuzumab (20 μg/ml) or MM-121 (10 μg/ml) alone, or their combinations for 24 hrs. All the cells were collected for analysis of cell cycle distributions by flow cytometry as described in the Methods. The bar graphs show the percentages of the cells at G1 phase for each sample relative to controls, defined as 100%. A, The combinations of trastuzumab and MM-121 as compared to trastuzumab significantly induced G1 arrest in SKBR3 cells. There was no significant difference between these two treatments in BT474 cells, which was very sensitive to the treatment of trastuzumab alone. B, The combinations of trastuzumab and MM-121 as compared to trastuzumab significantly induced G1 arrest in both SKBR3-pool2 and BT474-HR20 cells. Bars, SD. Statistical analyses were performed using data from three independent experiments. [file 1476-4598-12-134-S1.tiff]

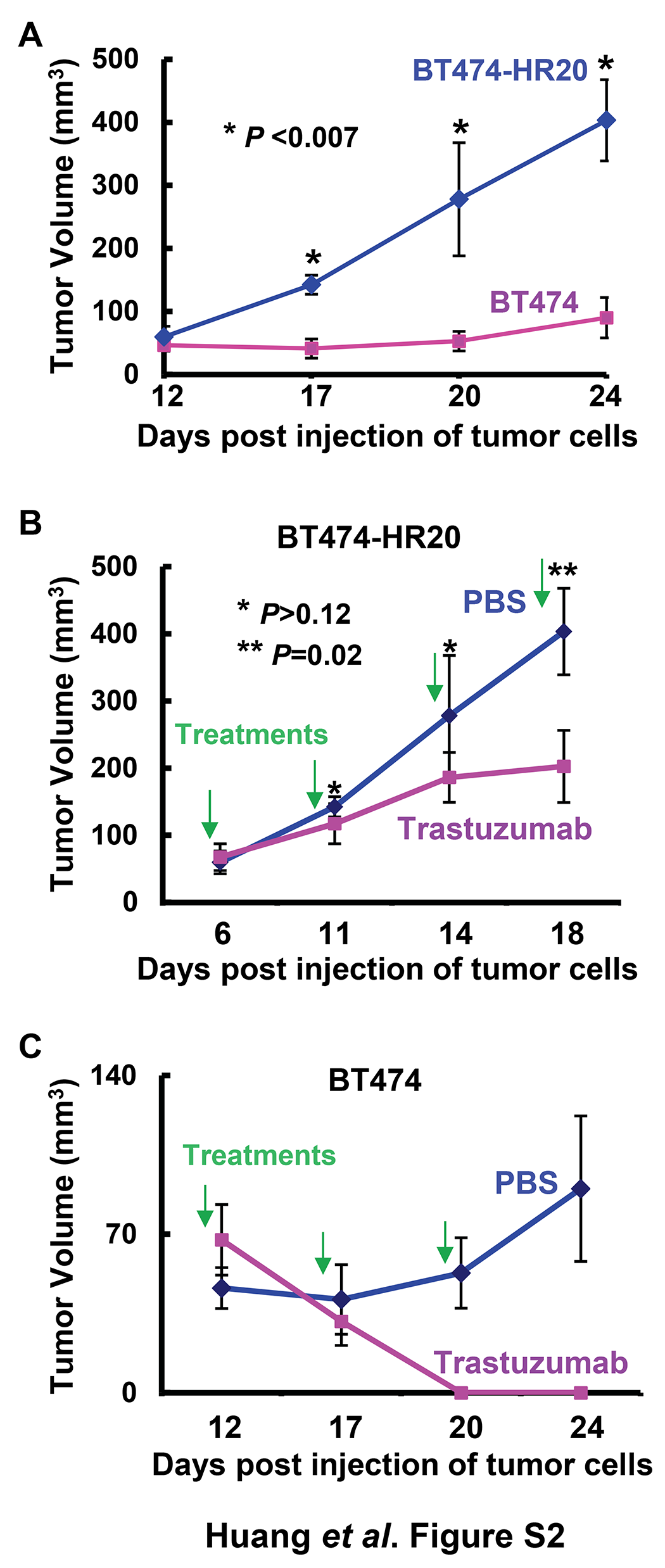

Supplement: Additional file 2: Figure S2 — Trastuzumab-resistant breast cancer cells show significant growth advantage as compared to their sensitive counterpart and retain their resistant phenotype in vivo. A, BT474 or BT474-HR20 cells were injected s.c into the flanks of 5-week-old female nude mice. Mice were checked for tumor formation three times per week. Tumor volume was calculated by the formula: volume = (length × width 2 )/2, and expressed as cubic millimeters. B & C, When tumor volumes reached ~65 mm3, the animals were treated with either control (PBS) or trastuzumab (20 mg/kg) four times. Tumors-derived from BT474-HR20 cells were still growing even in the presence of trastuzumab (B), whereas the tumors-derived from BT474 cells were no longer detectable after three doses of trastuzumab (C). Tumor volume was expressed as cubic millimeters (mean ± SE; n = 5/group). [file 1476-4598-12-134-S2.tiff]

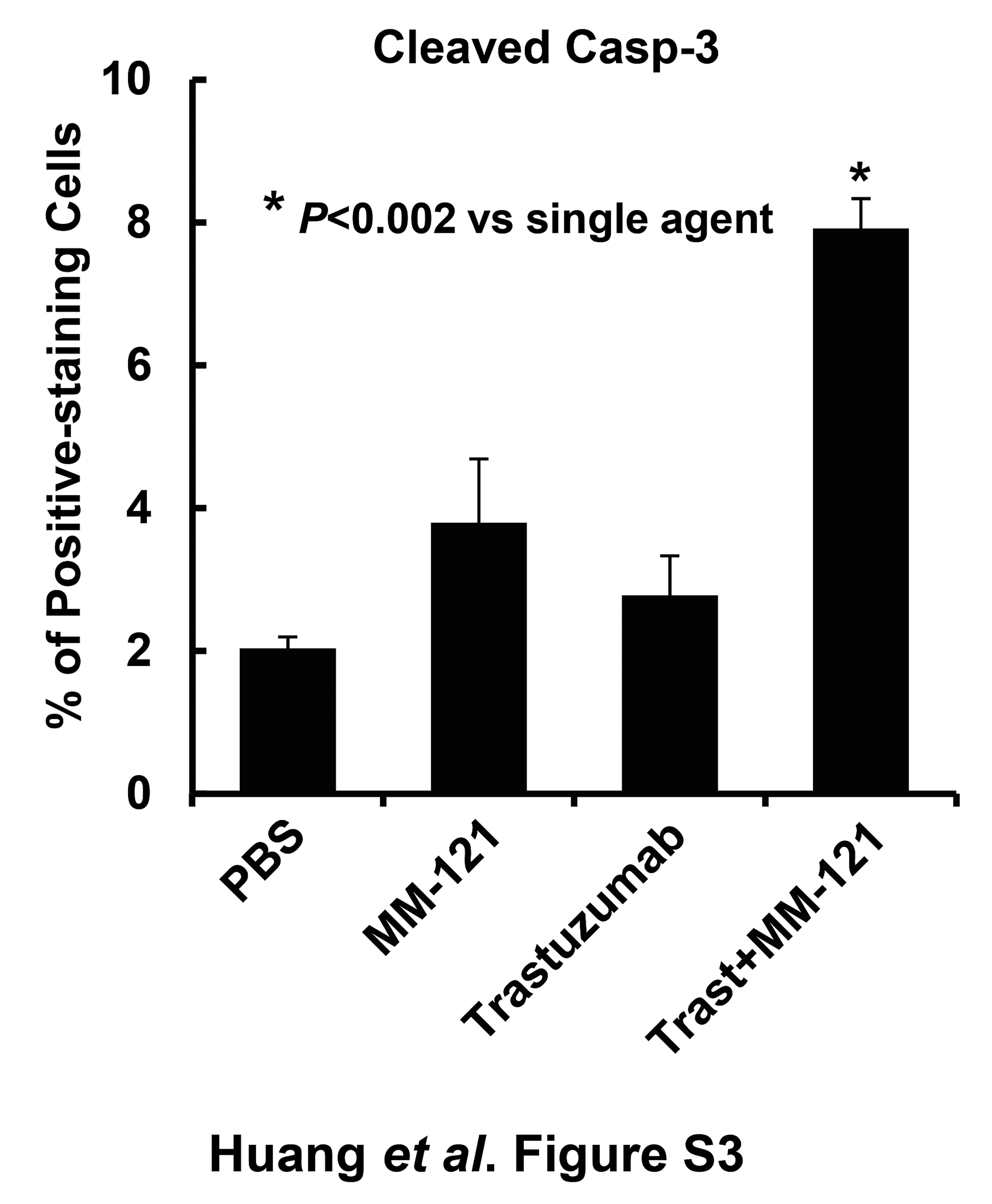

Supplement: Additional file 3: Figure S3 — Combinations of trastuzumab and MM-121 significantly increase the percentage of positive-staining cells with cleaved caspase-3 in vivo. The tumor IHC slides were observed by two independent personnel. The tumor cells with positive staining of cleaved caspase-3 were counted from three randomly selected areas (a total of 360 tumor cells were counted for each area) in each slide. The three areas were first identified by scanning the entire slide at ×10 magnification, and then the positive staining cells were counted at ×20 magnification using an Olympus B×40 Microscope. The bar graphs show the percentage of cells with positive staining for cleaved caspase-3 from each group. The combinatorial treated mice had significantly higher proportion of cells stained positive for cleaved caspase-3 than the control mice or single Ab treated mice, P < 0.002. [file 1476-4598-12-134-S3.tiff]
